# Auditory Cortex tACS and tRNS for Tinnitus: Single versus Multiple Sessions

**DOI:** 10.1155/2014/436713

**Published:** 2014-12-22

**Authors:** Laura Claes, Hannah Stamberger, Paul Van de Heyning, Dirk De Ridder, Sven Vanneste

**Affiliations:** ^1^Department of Translational Neuroscience, Faculty of Medicine, University of Antwerp, Campus Drie Eiken, Universiteitsplein 1, Wilrijk, 2610 Antwerpen, Belgium; ^2^Department of Surgical Sciences, Dunedin School of Medicine, University of Otago, Dunedin, New Zealand; ^3^Lab for Clinical & Integrative Neuroscience, School of Behavioral & Brain Science, University of Texas at Dallas, W. 1966 Inwood Road, Dallas, TX 75235, USA

## Abstract

Tinnitus is the perception of a sound in the absence of an external acoustic source, which often exerts a significant impact on the quality of life. Currently there is evidence that neuroplastic changes in both neural pathways are involved in the generation and maintaining of tinnitus. Neuromodulation has been suggested to interfere with these neuroplastic alterations. In this study we aimed to compare the effect of two upcoming forms of transcranial electrical neuromodulation: alternating current stimulation (tACS) and random noise stimulation (tRNS), both applied on the auditory cortex. A database with 228 patients with chronic tinnitus who underwent noninvasive neuromodulation was retrospectively analyzed. The results of this study show that a single session of tRNS induces a significant suppressive effect on tinnitus loudness and distress, in contrast to tACS. Multiple sessions of tRNS augment the suppressive effect on tinnitus loudness but have no effect on tinnitus distress. In conclusion this preliminary study shows a possibly beneficial effect of tRNS on tinnitus and can be a motivation for future randomized placebo-controlled clinical studies with auditory tRNS for tinnitus. Auditory alpha-modulated tACS does not seem to be contributing to the treatment of tinnitus.

## 1. Introduction

Tinnitus is the perception of a sound in the absence of external sound stimuli [1–3]. The prevalence of persistent idiopathic tinnitus is between 10 and 15 percent and it is often associated with a significant impact on quality of life [1, 4]. Tinnitus is generally believed to result from a peripheral defect (cochlear or nerve lesion) caused by triggers such as noise trauma, presbyacusis, or ototoxic medication, thereby inducing deafferentation [2, 5]. Currently it is hypothesized that changes in the central auditory pathway (nucleus cochlearis, colliculus inferior, thalamus, and auditory cortex) as well as in nonauditory brain structures play a major role in the generation and maintaining of tinnitus [6–9], filling in the missing information from the auditory periphery [10]. This fits in a network model of tinnitus [11], in which the unified tinnitus percept is the result of multiple parallel partially overlapping and dynamically changing networks [12, 13], each with its own oscillatory signature and each representing a separate clinical aspect of the tinnitus, such as loudness, annoyance, depression, and lateralization [14].

Several reviews suggest that due to peripheral hearing loss a deficient auditory input induces maladaptive neuroplasticity changes, with [15] or without [16] tonotopic map reorganization [5, 17–19]. Some recent studies have suggested the “thalamocortical dysrhythmia” model as a possible pathophysiological mechanism for tinnitus [7, 20, 21]. It is suggested in multiple studies that the thalamus plays an important role as an independent pacemaker for cortical rhythms [22]. In healthy subjects, neural alpha oscillations have been identified as the dominant frequency in the auditory cortex during rest [20]. However in tinnitus patients, the auditory cortex shows increased gamma band activity [20] associated with reduced activity in the alpha frequency range [23]. Thalamocortical dysrhythmia implies that auditory deafferentation of the thalamus with or without [24] hearing loss induces slow theta oscillations (4–8 Hz) in the deafferented thalamocortical columns [21]. As a result, reduced lateral inhibition causes high-frequency gamma oscillations to surround these theta oscillations [21]. Recently the “synchronization by loss of inhibition model” was introduced. It postulates that the marked alpha activity in normal human auditory regions reflects a state of functional inhibition [7, 25]. This inhibition prevents cells from spontaneous synchronization, which on a clinical level could result in a phantom perception. Downregulation of inhibition in deafferented regions expressed by reduced alpha activity may therefore lead to enhanced spontaneous synchronization, expressed by augmented gamma activity. Lorenz et al. [26] also emphasize the inverse relationship between alpha and gamma activity.

Neuromodulation can be used to modulate these plastic changes. Several studies confirm the value of transcranial direct current stimulation (tDCS) in modulating tinnitus loudness and annoyance [27–34]. Currently, two new mechanisms of transcranial stimulation have been developed: transcranial alternating current stimulation (tACS) and transcranial random noise stimulation (tRNS). Both methods presumably have a different working mechanism than tDCS. tACS is similar to tDCS, but with alternating current (because of constant polarity changes), instead of direct current. Based on positive study results with tACS in other cerebral functions [35–38], we presume that tACS could possibly contribute to the treatment of tinnitus. Furthermore, it is a promising technique because in addition to the location, the stimulation frequency can be altered. By using alpha-modulated tACS on the auditory cortex we try to influence the decreased alpha activity seen in tinnitus patients. Previous studies demonstrated that tACS can increase the alpha activity in the stimulated cortex [39].

The effect of tACS on the auditory cortex has not been investigated in a clinical study yet. tRNS is a modified version of tACS, with a white noise configuration in a Gaussian distribution between 0.1 and 640 Hz [40]. There is electrophysiological evidence of the feasibility of tRNS to modulate and desynchronize specific oscillatory brain activity [37, 40–43]. tNRS is easy to use in a randomized setting because a higher skin perception threshold reduces the likelihood of a burning or itching sensation [44, 45].

The effect of tACS and tRNS after multiple sessions could possibly differ from that after single session stimulation, analogous to what has been shown for TMS [46–48]. Electrical stimulation might strengthen synaptic connections through a mechanism similar to long-term potentiation [46], similar to what has been claimed for TMS on the auditory cortex [47]. Therefore multiple sessions of electrical stimulation could obtain a similar effect to that of TMS.

In this study, we aim to evaluate the efficacy of alpha-modulated tACS and tRNS on tinnitus loudness and tinnitus annoyance, while comparing the effect of single session versus multiple sessions of stimulations targeting the auditory cortex.

## 2. Methods

### 2.1. Patients

228 patients (167 males and 61 females) diagnosed with chronic nonpulsatile tinnitus (>1 year) participated in this study at the TRI tinnitus clinic of the Antwerp University Hospital in Belgium. The mean age and mean tinnitus duration were 51.40 and 6.60 years, respectively. Sixty-nine patients presented with pure tone tinnitus, 121 with narrow band noise tinnitus, and 107 with both pure tone and narrow band noise tinnitus, while 83 had unilateral tinnitus, 144 had bilateral tinnitus, and for 1 patient the lateralization was unknown. All subjects were fluent in Dutch, permitting understanding the informed consent. Drug therapy was stable immediately prior to treatment and during the treatment. Patients that took specific medication (i.e., amine metabolism drugs: citalopram, amphetamine, L-dopa, sulpiride, and pergolide; amino acid metabolism drugs: lorazepam, rivastigmine, dextromethorphan, and D-cycloserine; and voltage-sensitive channel blockers: carbamazepine and flunarizine) that could modify the stimulation effect were excluded [49]. The study was in accordance with the ethical standards of the Helsinki declaration. Data were collected into a and retrospectively analyzed. This study was approved by the institutional ethics committee of the Antwerp University Hospital.

### 2.2. Design

Patients were retrospectively retrieved from a database and included in one of the four study arms, being “single session tACS” (*N* = 40), “repeated tACS” (*N* = 11), “single session tRNS” (*N* = 167), and “repeated tRNS” (*N* = 10). Each session consisted of 20 minutes of stimulation with tACS or tRNS. Repeated sessions were given twice per week (Monday–Thursday or Tuesday–Friday) with a total of 8 stimulations delivered during 4 weeks.

### 2.3. EEGs

EEGs (Mitsar, Nova Tech EEG, Inc., Mesa) were obtained one week before the tACS stimulation in a fully lighted room with each participant sitting upright in a comfortable chair with closed eyes. The EEG was sampled with 19 electrodes (Fp1, Fp2, F7, F3, Fz, F4, F8, T7, C3, Cz, C4, T8, P7, P3, Pz, P4, P8, O1, and O2) in the standard 10–20 International placements referenced to linked lobes. Impedance was checked to remain below 5 kΩ. Data was collected for 100 2-s epochs, the sampling rate = 1024 Hz, and band passed 0.15–200 Hz. Data was resampled to 128 Hz and band-pass filtered (fast Fourier transform filter) to 2–44 Hz. This data was transposed into Eureka! Software (Congedo, 2002) and plotted and carefully inspected manually for artifact. All episodic artifacts including eye blinks, eye movements, teeth clenching, body movement, and ECG artifacts were removed from the stream of the EEG.

### 2.4. tACS

Alternating current was transmitted by a saline-soaked pair of surface sponges (35 cm²) and delivered by specially developed, battery-driven, constant current stimulator with a maximum output of 10 mA (NeuroConn; http://www.neuroconn.de/). For each patient receiving tACS, one electrode was placed over the left auditory cortex and one was placed on the right auditory cortex as determined by the International 10/20 Electroencephalogram System corresponding to T3 and T4, respectively. The frequency of the tACS was set to the individual alpha frequency (IAF). The AC current was initially increased in a ramp-like fashion over several seconds (10 s) until reaching 2.0 mA. In tACS, stimulation was maintained for a total of 20 min. To determine the frequency of stimulation, the IAF peak was identified according to literature guidelines [48]. This individual alpha frequency peak was defined as the frequency within the range of 6–13 Hz, range of the EEG spectrum showing maximum power for the electrodes T3 and T4.

### 2.5. tRNS

The tRNS consisted of an alternating current of 2.0 mA intensity with a 0 mA offset applied at random frequencies. The frequencies ranged from 0.1 to 100 Hz, that is, low frequency tRNS. Similar to tDCS or tACS the current was transmitted by a saline-soaked pair of surface sponges (35 cm²) and delivered by specially developed, battery-driven, constant current stimulator with a maximum output of 10 mA (NeuroConn; http://www.neuroconn.de/). For each patient receiving tRNS, one electrode was placed on the T3 and one was placed on T4 as determined by the International 10/20 Electroencephalogram System. The AC current was initially increased in a ramp-like fashion over several seconds (10 s) until reaching 2.0 mA. In tRNS, stimulation was maintained for a total of 20 min.

### 2.6. Evaluation

To evaluate the effects of each intervention, we measured changes in tinnitus loudness (“How loud is your tinnitus? 0 = no tinnitus and 10 = as loud as imaginable”) and tinnitus annoyance (“How annoying is your tinnitus? 0 = not annoying and 10 = suicidal annoying”). These numeric rating scales (NRS) were asked before (pre) and directly after (post) stimulation.

### 2.7. Statistical Analyses

Collected data were analyzed using “SPSS 20.0” with paired two-sample *t*-test comparing before and directly after stimulation tinnitus loudness and tinnitus annoyance for, respectively, a single session of tRNS, a single session of tACS, multiple sessions of tRNS, and multiple sessions of tACS.

An independent *t*-test was conducted comparing a single session with multiple sessions of, respectively, tRNS and tACS for difference score (= prestimulation − poststimulation) on tinnitus loudness and tinnitus annoyance. The sample size for the patients who received a single session in comparison to multiple sessions was larger. This unbalanced design may lead to overpowering for the single session group. We conducted a resampling analysis (i.e., bootstrapping) in which we draw random participants from the multiple session group and tested if the same results are obtained in this randomly selected smaller group. This process was repeated 1000 times.

An independent *t*-test was conducted comparing tRNS and tACS for, respectively, a single session and multiple sessions for difference score (= prestimulation – poststimulation) on tinnitus loudness and tinnitus annoyance. As the sample size for the patients who received a tRNS and tACS was different, the statistical design is unbalanced which may lead to overpowering for the single group. Therefore, we conducted a resampling analysis (i.e., bootstrapping) in which we draw random participants from the multiple session group and tested if the same results could be obtained in this randomly selected smaller group. This process was repeated 1000 times.

Furthermore, we included an additional analysis where we control specific tinnitus characteristics (type: narrow band noise versus pure tone; lateralization: unilateral or bilateral tinnitus). We only applied this for the single session of tACS and tRNS because not an adequate number of subjects were included in the multiple sessions to control these variables. We conducted an independent *t*-test with the difference score (prestimulation – poststimulation) as dependent variable and tinnitus characteristics as independent variable.

## 3. Results

### 3.1. Single Sessions

A paired *t*-test for the tRNS data revealed a significant difference on both tinnitus loudness (*t*(166) = 3.76, *P* < .001) and tinnitus annoyance (*t*(166) = 3.16, *P* < .01) demonstrating that after stimulation a mean suppression effect was obtained for tinnitus loudness from 6.62 (Sd = 1.91) to 6.24 (Sd = 2.12) and for tinnitus annoyance from 6.17 (Sd = 2.21) to 5.85 (Sd = 2.38).

**Figure 1:**
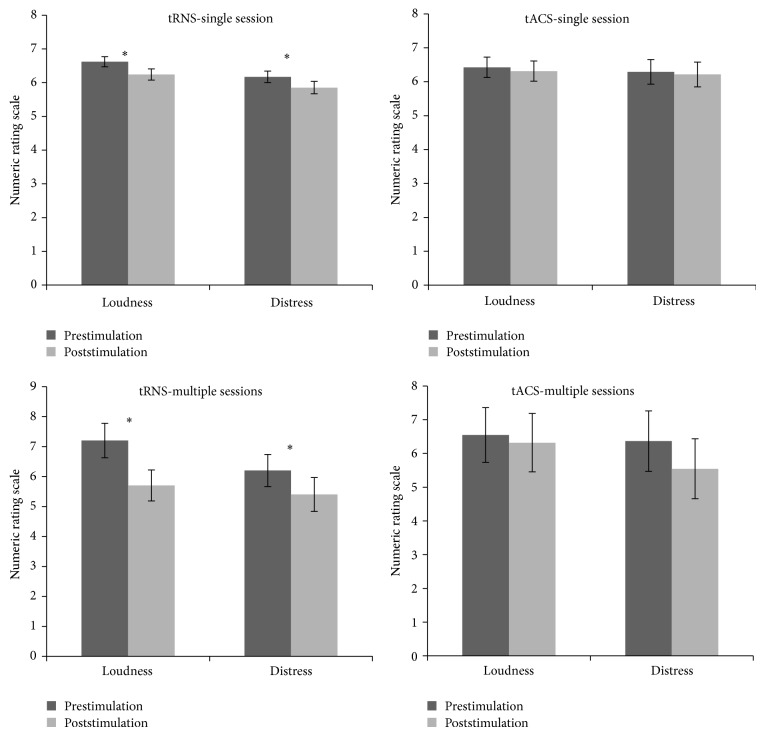
Overview of the obtained results for tinnitus loudness and annoyance after a single session or multiple sessions of random noise stimulation (tRNS) or alternating current stimulation (tACS).

A similar analysis for tACS revealed no significant effect on both tinnitus loudness (*t*(39) = 1.85, *P* = .07) and tinnitus annoyance (*t*(39) = 1.37, *P* = .18) demonstrating that after stimulation no suppression effect was obtained for tinnitus loudness from 6.43 (Sd = 1.89) to 6.31 (Sd = 1.88) and for tinnitus annoyance from 6.29 (Sd = 2.29) to 6.21 (Sd = 2.31). See Figure 1 for overview.

### 3.2. Multiple Sessions

After multiple sessions of tRNS a significant effect was demonstrated on both tinnitus loudness (*t*(9) = 4.03, *P* < .01) and tinnitus annoyance (*t*(9) = 3.21, *P* < .05) a mean suppression effect for tinnitus loudness from 7.20 (Sd = 1.81) to 5.70 12 (Sd = 1.64) and for tinnitus annoyance from 6.20 (Sd = 1.69) to 5.40 (Sd = 1.78).

Multiple sessions of tACS revealed did not lead to a significant effect on both tinnitus loudness (*t*(10) = .81, *P* = .44) and tinnitus annoyance(*t*(10) = 1.63, *P* = .13). Before stimulation, the mean tinnitus loudness and mean tinnitus annoyance were 6.55 (Sd = 2.70) and 6.36 (Sd = 2.98), respectively. After the stimulation sessions the mean tinnitus loudness and mean tinnitus annoyance were 6.32 (Sd = 2.88) and 5.54 (Sd = 2.94). See Figure 1 for overview.

### 3.3. Single versus Multiple Sessions

A comparison was made between a single session and multiple sessions of tRNS on the difference scores (= prestimulation − poststimulation) and revealed a significant effect indicating an increased suppression effect for multiple sessions (M = 1.50, Sd = 1.18) in comparison to a single session (M = .38, Sd = 1.30) for tinnitus loudness (*t*(175) = −2.67, *P* < .01). However, no significant effect was obtained for tinnitus annoyance (*t*(175) = −1.16, *P* = .25). A single session had a main effect of .32 (Sd = 1.30) and multiple sessions had a main effect of .80 (Sd = .79). A bootstrap analysis further confirmed our findings and revealed a stable effect (bias = .02, S.E. = .39, *P* = .01) for tinnitus loudness, while for tinnitus annoyance no significant effect was obtained (bias = .02, S.E. = .27, *P* = .08).

A comparison between a single session and multiple sessions of tACS revealed no significant effect for tinnitus loudness (*t*(49) = −.62, *P* = .54): a single session had a mean effect of .11 (Sd = .38) and multiple sessions had a mean effect of .22 (Sd = .93). However, for tinnitus annoyance a significant effect was obtained (*t*(49) = −.62, *P* = .54) demonstrating an increased suppression effect for multiple sessions (M = .81, Sd = 1.66) in comparison to a single session (M = .08, Sd = .35). However, a bootstrap analysis further indicated that, for both tinnitus loudness (bias = .01, S.E. = .29, *P* = .54) and tinnitus annoyance, no significant effect was obtained (bias = .02, S.E. = .27, *P* = .34). This suggests that the initially obtained effect for the tinnitus annoyance might by due to overpowering of the sample size for a single session. See Figure 1 for overview.

### 3.4. Comparison between tACS and tRNS

A between stimulation comparison for a single session revealed a significant effect for tinnitus loudness (*t*(205) = 2.27, *P* < .05) and for tinnitus annoyance (*t*(205) = 2.11, *P* < .05) revealing that tRNS had a significantly larger suppression effect (loudness: M = .38, Sd = 1.30; annoyance: M = .32, Sd = 1.30) compared to tACS (loudness: M = .11, Sd = .38; annoyance: M = .08, Sd = .35). A bootstrap analysis further confirmed our findings and revealed a stable effect (bias = −.002, S.E. = .11, *P* < .05) for tinnitus loudness and for tinnitus annoyance (bias = .0032, S.E. = .11, *P* < .05).

A similar analysis was obtained between tACS and tRNS for multiple sessions demonstrating a significant effect (*t*(19) = 2.76, *P* < .05) for tinnitus loudness, but no significant effect was obtained for tinnitus annoyance (*t*(19) = −.03, *P* = .97). For tinnitus loudness it was revealed that multiple sessions of tRNS had a larger suppression effect (M = 1.50, Sd = 1.18) than tACS (loudness: M = .28, Sd = .93). Tinnitus annoyance had a mean suppression effect of .80 (Sd = .79) for tRNS and .81 (Sd = 1.66) for tACS. A bootstrap analysis further confirmed our findings and revealed a stable effect (bias = .02, S.E. = .46, *P* < .05) for tinnitus loudness, while for tinnitus annoyance no significant effect was obtained (bias = .03, S.E. = .53, *P* = .67). See Figure 1 for overview.

### 3.5. Tinnitus Characteristics

An additional analysis including tinnitus characteristics revealed no significant effects for both tinnitus loudness and tinnitus annoyance (*P* > .23).

## 4. Discussion

In this study, we aimed to compare the effects of single versus multiple sessions of alpha-modulated tACS and tRNS targeting the auditory cortex for tinnitus loudness and annoyance. Our results demonstrate that tRNS of the auditory cortex improved both tinnitus loudness and annoyance. This was the case for both single and multiple sessions of tRNS. Alpha-modulated tACS, however, did not show any significant effect. In addition, it was demonstrated that multiple sessions of tRNS exerted an increased suppression effect on the tinnitus loudness in comparison to a single session of tRNS. This effect was not found for tinnitus annoyance.

Auditory alpha-modulated tACS seems to have no effect on tinnitus analogous to what has been shown for single sessions of tACS on the dorsolateral prefrontal cortex [30]. Even with multiple sessions, tACS did not improve tinnitus. This suggests that tACS at the individual alpha frequency is not beneficial for tinnitus, when applied on neither the DLPFC nor the auditory cortex.

The results of this study show a significant effect of auditory stimulation with tRNS on tinnitus loudness and annoyance. Another study of this research group (Vanneste et al.) made a comparison between the effects of single sessions tDCS, alpha-modulated tACS, and tRNS on the auditory cortex [50]. This study confirms that tRNS on the auditory cortex has a greater suppressive effect on tinnitus loudness and annoyance than tACS (and tDCS).

The results also suggest that tRNS and tACS might have a different working mechanism with a different impact on the stimulated brain tissue. Terney et al. were the first to introduce tRNS as a novel method of transcranial electrical stimulation [41]. They hypothesize that tRNS might interfere with ongoing oscillations and neuronal activity in the brain, which in turn could translate to an increase in cortical excitability. They hypothesize that the neuroplasticity effects of tRNS are similar to the anodal tDCS after-effect, with the benefits of tRNS being not polarity dependent. The fact that tRNS does not work in a polarity dependent way could ensure that it does not induce the homeostatic phenomena of ion neuron channels [41]. With tDCS, the neurons are subjected to a constant electrical field. Therefore the channels on the neuronal membrane adapt themselves and return to their original resting state. This does not form a problem with tRNS because of the constantly changing electrical field. Fertonani and coworkers hypothesize that tRNS induces a temporal summation of neuronal activity if the time constant of the neuron is sufficiently long to allow the summation of two stimuli presented in close sequence [40]. Thus, stimulated neurons would approach their response threshold quicker. A third mechanism that has been put forward in different studies is the phenomenon of “stochastic resonance.” Moss et al. [51] showed that nonlinear systems, like our brain, can use noise to modulate existing neuronal signals. According to this principle we hypothesize that the effect of tRNS on tinnitus could be explained by the capacity of tRNS to interrupt the pathological synchronized neuronal activity we see in tinnitus patients.

Additionally, we demonstrated that repetitive sessions of auditory cortex targeting tRNS could strengthen the effect on tinnitus loudness, yet they had no additional effect on tinnitus annoyance. This confirms that auditory cortex tRNS mainly modulates tinnitus loudness and not the annoyance, which is mainly mediated in the resting state via the cingulate, insula, and medial temporal lobe structures [52–54].

This study should be seen as a pilot study and should be followed by future studies exploring the effect of tACS and tRNS on tinnitus. In this preliminary study it is demonstrated that it could be useful to further study the effects of tRNS on the auditory cortex. Further investigation with alpha-modulated tACS on the auditory cortex does not seem beneficial.

The design of this study has some restrictions. Our study population was retrospectively selected and was not placebo controlled. The fact that participants cannot tell the difference between alternating stimulation and noise stimulation suggests that the effect obtained by noise stimulation is presumably not placebo related. Furthermore, in this study, possible influencing variables (e.g., age, sex, tinnitus characteristics, etc.) are not yet taken into account. It should also be noted that noninvasive electrical stimulation is not random and is often distorted by a lot of variables (e.g., stimulation parameter, location, impedance, etc.). To evaluate the effect of tRNS as a potential treatment for tinnitus in daily practice, large randomized placebo-controlled studies should be undertaken, also investigating possible variables and confounders.

In summary, in this preliminary study we explored the effect of single and repetitive sessions of auditory cortex alpha-modulated tACS and tRNS for the treatment of tinnitus. The results showed that both single session and repetitive tRNS have a significant effect on tinnitus loudness and annoyance. Multiple sessions of tRNS increase the effect on tinnitus loudness but have no additional effect on tinnitus annoyance. Individually adjusted alpha-modulated tACS on the auditory cortex did not show any significant effect, neither in single nor in repetitive sessions. To evaluate the effect of tRNS as a potential treatment for tinnitus, large randomized placebo-controlled studies should be undertaken.
